# Skin fibroblasts of patients with geleophysic dysplasia due to *FBN1* mutations have lysosomal inclusions and losartan improves their microfibril deposition defect

**DOI:** 10.1002/mgg3.844

**Published:** 2019-07-27

**Authors:** Pasquale Piccolo, Valeria Sabatino, Pratibha Mithbaokar, Elena Polishchuk, John Hicks, Roman Polishchuk, Carlos A. Bacino, Nicola Brunetti‐Pierri

**Affiliations:** ^1^ Telethon Institute of Genetics and Medicine Pozzuoli Italy; ^2^ Department of Translational Medicine Federico II University of Naples Naples Italy; ^3^ Department of Pathology Baylor College of Medicine Houston Texas; ^4^ Department of Molecular and Human Genetics Baylor College of Medicine Houston Texas; ^5^ Present address: Center for Molecular and Cellular Bioengineering Technische Universität Dresden Dresden Germany

**Keywords:** extracellular matrix, geleophysic dysplasia, intracytoplasmic inclusions, losartan

## Abstract

**Background:**

Geleophysic dysplasia (GPHYSD) is a disorder characterized by dysmorphic features, stiff joints and cardiac involvement due to defects of TGF‐β signaling. GPHYSD can be caused by mutations in *FBN1*, *ADAMTLS2*, and *LTBP3* genes.

**Methods and Results:**

Consistent with previous reports, we found intracellular inclusions of unknown material by electron microscopy (EM) in skin fibroblasts of two GPHYSD individuals carrying *FBN1* mutations. Moreover, we found that the storage material is enclosed within lysosomes and is associated with the upregulation of several lysosomal genes. Treatment of GPHYSD fibroblasts carrying *FBN1* mutations with the angiotensin II receptor type 1 inhibitor losartan that inhibits TGF‐β signaling did not reduce the storage but improved the extracellular deposition of fibrillin‐1 microfibrils.

**Conclusion:**

Losartan is a promising candidate drug for treatment of GPHYSD due to *FBN1* defects.

## 1 INTRODUCTION

Geleophysic dysplasia (GPHYSD1, MIM231050; GPHYSD2, MIM614185; GPHYSD3, MIM617809) is a connective tissue disorder presenting with short stature, small hands and feet, cardiac valvular disease, hepatomegaly, joint contractures, and thickened skin (Rosser, Wilkinson, Hurst, McGaughran, & Donnai, 1995; Spranger, Gilbert, Tuffli, Rossiter, & Opitz, 1971; Vanace, Friedman, & Wagner, 1960). Although initially suspected to be a lysosomal storage disorder (Lipson, Kan, & Kozlowski, 1987; Wraith, Bankier, Chow, Danks, & Sardharwalla, 1990), GPHYSD was later recognized to be caused by mutations in genes encoding components of the extracellular matrix (ECM) including a disintegrin and metalloproteinase with thrombospondin motifs like 2 (ADAMTSL2, MIM612277) (GPHYSD1) (Le Goff et al., 2008), fibrillin 1 (*FBN1*, MIM134797) (GPHYSD2) (Le Goff et al., 2011), and latent transforming growth factor β (TGF‐β) binding protein 3 (*LTBP3*, MIM 602090) (GPHYSD3) (McInerney‐Leo et al., 2016). Transforming Growth Factor‐β (TGF‐β) is sequestered in an inactive and latent form by interactions with several ECM proteins and ordered polymers of fibrillin bind latent TGF‐β maintaining TFG‐β biologically inactive. Defects in GPHYSD genes perturb TGF‐β signaling and result in altered deposition of ECM (Le Goff et al., 2011, 2008). Mutations in *FBN1* result in Marfan syndrome (MFS) (Robinson et al., 2006) but domain‐specific *FBN1* mutations have been found in other connective tissue disorders including Weill‐Marchesani syndrome (Faivre et al., 2003), stiff skin syndrome (Loeys et al., 2010), isolated ectopia lentis (Lonnqvist et al., 1994), and acromicric dysplasia (Le Goff et al., 2011), in addition to GPHYSD. In GPHYSD patients, heterozygous *FBN1* mutations are all located in exons 41 and 42 encoding for TGF‐β‐binding protein‐like domain 5 (TB5) (Le Goff et al., 2011).

In the present study, we report the ultrastructural observation of a storage phenotype in skin fibroblasts of GPHYSD patients carrying *FBN1* mutations. Moreover, we found that the storage material is enclosed within lysosomes and is associated with upregulation of several lysosomal genes. Finally, we evaluated the efficacy of the angiotensin II receptor type 1 inhibitor losartan that inhibits TGF‐β signaling for correction of ECM deposition defect in fibroblasts of a GPHYSD patient carrying a *FBN1* mutation.

## 2 CASE REPORTS

Clinical features of the subjects 1 and 3 from whom skin fibroblasts were obtained are summarized in Table 1. Subjects 1 and 2 are siblings and both presented with short stature and brachydactyly. Subject 1 is currently a 27‐year‐old female and she presented with short stature and pulmonic valve stenosis. She underwent a valvotomy at the age of 13 years. On physical exam at the age of 18 years, her height was 126.6 cm (below the 3rd centile) with an arm span of 116 cm. She had pleasant appearing facies, short arms and brachydactyly with a trigger left thumb. II/VI diastolic murmur was heard over the left sternal border. She delivered an affected son preterm at 33 3/7 weeks gestation. At 6 months, his corrected length was at the 10th centile but by the age of 18 months, his linear growth decelerated, and his height had fallen below the 3rd centile. His physical exam was remarkable for brachydactyly with significant shortening of the middle phalanx of II and V digits. Subject 2 is currently a 25‐year‐old male who also presented short stature and brachydactyly. At the age 10 years of his last evaluation, his height was 117.7 cm (below the 3rd centile) with an arm span 104 cm. He had short limbs, brachydactyly, and pleasant facies. Brother, sister and sister's son were cognitively normal. Both sibs were born to unaffected parents, thus suggesting gonadal mosaicism. Skin fibroblasts were not available for subject 2.

**Table 1:** Summary of clinical, molecular, and ultrastructural findings

	Subject		
	1	2^a^	3^b^
Age at last evaluation (years)	10	13	1.8
Gender	F	M	M
Ethnicity	Hispanic	Hispanic	Hispanic/ African American
Clinical features			
Short stature	+	+	+
Short neck	−	−	−
Laryngeal or tracheal stenosis	−	−	+
Chronic respiratory infections	−	−	−
Cardiac valve disease	+	−	+
Hepatomegaly	−	−	−
Motor delay	−	−	+
Skin stiffness	−	−	+
Small hands/feet	+	+	+
Joint contractures	−	−	+
Tiptoe gait	−	−	−
J‐shaped sella	+	+	+
Short small tubular bones	+	+	−
*FBN1* variant	c.5284G>A p.(Gly1762Ser)	N.A.	c.5117G>A p.(Cys1706Tyr)
EM inclusions	+	N.A.	+

Note

M, male; F, female; N.A., not available; EM, electron microscopy. *FBN1* NCBI Reference Sequences were NC_000015.10 for the gene, NM_000138.4 for the mRNA, and NP_000129.3 for the protein.

a Sibling of subject 1.

b Previously described in Sule et al. (2013).

Clinical findings of subject 3 were previously reported [as subject 6 by Sule et al. (2013)]. Upon parental consent, skin biopsies were performed prior to genetic testing. Sanger sequencing of exons 41 and 42 of *FBN1* identified heterozygous missense mutations which were previously reported (Le Goff et al., 2011) (Table 1).

## 3 MATERIALS AND METHODS

Genomic DNA was extracted from peripheral blood by standard procedures. PCR primers for *FBN1* amplification were previously described (Le Goff et al., 2011, 2008, 2012). Sanger sequencing was performed by PRIMM. *FBN1* NCBI Reference Sequences were NC_000015.10 for the gene, NM_000138.4 for the mRNA, and NP_000129.3 for the protein.

### 3.1 Cell culture studies

Fibroblast primary cultures were established from skin biopsy samples from GPHYSD, MS and MFS cases, and from healthy volunteers. Cells were cultured according to standard procedures and maintained in Dulbecco's Modified Eagle Medium (DMEM) (EuroClone) with 10% Fetal Bovine Serum (FBS) (Euroclone) and penicillin/streptomycin (Sigma‐Aldrich), in a humidified atmosphere containing 5% CO_2_ at 37°C. Cells were treated for 1 week (1 ng/ml) or 48 hr (10 ng/ml) with mature TGF‐β1 (Sigma‐Aldrich) or vehicle (4mM HCl‐0.1% human serum albumin). Losartan potassium salt (Sigma‐Aldrich) was dissolved into dimethyl sulfoxide (DMSO) (Sigma‐Aldrich) and used at the concentration of 200 μM for 14 days. At confluency, fibroblasts were trypsinized and resuspended in glutaraldehyde for electron microscopy (EM) analysis.

### 3.2 EM and Immuno‐EM

For immuno‐EM analysis of LAMP‐1 distribution in fibroblasts, cells were fixed with the mixture of 4% paraformaldehyde (PFA) and 0.05% glutaraldehyde for 10 min at room temperature, then washed with 4% PFA to remove the residual glutaraldehyde and fixed again with 4% PFA for 30 min at room temperature. Next, cells were incubated with the blocking/permeabilizing mixture (0.5% Bovine Serum Albumin, 0.1% saponin, 50 mM NH_4_Cl) for 30 min and subsequently with the primary monoclonal antibody against LAMP1 (Developmental Studies Hybridoma Bank; cat.# H4A30), diluted 1:500 in blocking/permeabilizing solution. On the following day, cells were washed and incubated with the secondary antibody, the anti‐rabbit Fab’ fragment coupled to 1.4‐nm gold particles (diluted 1:50 in blocking/ permeabilizing solution) for 2 hr at room temperature. Specimens were then postfixed as previously described (Polishchuk & Polishchuk, 2013). After dehydration, the specimens were embedded in epoxy resin and polymerized at 60°C for 72 hr. Thin 60 nm sections were cut at the Leica EM UC7 microtome. EM images were acquired from thin sections using a FEI Tecnai‐12 electron microscope equipped with a VELETTA CCD digital camera (FEI, Eindhoven, The Netherlands). To count the number of lysosomes within the cells, EM images were acquired at the same magnification. Thirty‐five cells (for each sample) randomly distributed on single thin section were taken in consideration. Lysosomes were manually counted on EM images.

### 3.3 Gene expression analyses

Total RNA was extracted from liver tissue in TRIzol reagent (Invitrogen) using RNeasy kit (Qiagen). RNA was reverse transcribed using a first‐strand complementary deoxyribonucleic acid kit with random primers according to the manufacturer's protocol (Applied Biosystems). The qRT‐PCR reactions were performed using SYBR Green Master Mix in a Roche Light Cycler 480 system (Roche). Primer sequences are available in Supplementary Table 1 in Data S1. PCR conditions were as follows: preheating, 5 min at 95°C; 40 cycles of 15 s at 95°C, 15 s at 60°C, and 25 s at 72°C. Quantification results were expressed in terms of cycle threshold (Ct). Ct values were averaged for each technical duplicate. For expression analysis, *HPRT1* and *B2M* housekeeping genes were used as endogenous controls (reference markers) using LightCycler 480 software version 1.5. Differences between mean Ct values of tested genes and those of the reference gene were calculated as ΔCt gene = Ct gene − Ct reference. WT1 sample was used as calibrator and relative fold increase in expression levels was determined as E^−ΔΔCt^, E being primer efficiency.

### 3.4 Immunofluorescence stainings

FBN1 and COL1A1 immunofluorescence on fibroblasts were performed with anti‐FBN1 (Millipore; cat.# MAB1919) and anti‐COL1A1 (Millipore; cat.#AB758) antibodies as previously described (Piccolo et al., 2014). Confocal images were obtained using LSM710 confocal laser scanning microscope and ZEN2008 software (Carl Zeiss). Each experiment was performed in duplicate and at least five images per experiment were analyzed for each staining using ImageJ software.

### 3.5 Statistical analyses

Statistical significance was computed using the Student's two tail test or one sample two tail test. A *p* value < 0.05 was considered statistically significant.

## 4 RESULTS

**Figure 1:**
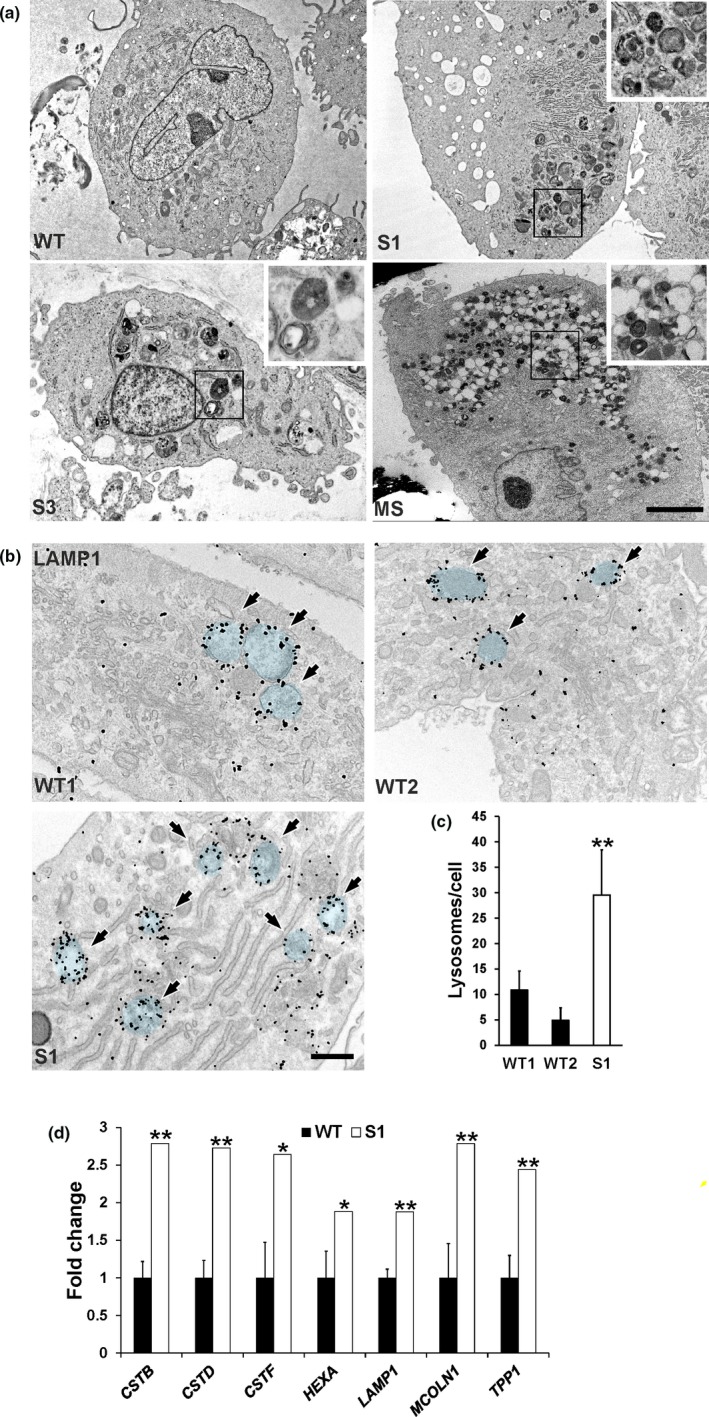
Lysosomal storage inclusions in GPHYSD fibroblasts. (a) Representative EM images of cultured skin fibroblasts from patients with GPHYSD [subject 1 (S1) and subject 3 (S3], Myhre syndrome (MS) carrying the p. (Arg496Cys) variant in *SMAD4* gene showing intracytoplasmic multilamellar and electron‐dense inclusions in GPHYSD and MS fibroblasts. Cell from healthy subject (WT) is shown as control. Scale bar: 5 µm. (b) LAMP‐1 immunogold staining in subject 1 fibroblasts (S1) showing storage within LAMP‐1 decorated lysosomal vesicles highlighted in blue and indicated by arrows. Cells from two healthy subjects (WT1 and WT2) are shown as controls. Scale bar: 500 nm. (c) LAMP‐1–positive vesicle count in GPHYSD fibroblasts (subject 1, S1) compared to two WT controls (WT1 and WT2). Averages ± *SEM* are shown; **p < 0.005 versus WT1 and WT2; *t *test. (d) qPCR for lysosomal genes in GPHYSD fibroblasts from subject 1 compared to WT controls (*n* = 4). Averages ± *SEM* are shown; *p < 0.05, **p < 0.005; one sample *t *test

Primary fibroblasts from skin biopsies (subjects 1 and 3) analyzed by EM showed intracellular inclusions with lamellar structure appearance, electron‐dense storage material, and large apparently empty vesicles (Figure 1a). Similar intracellular structures were also found in skin fibroblasts from three patients carrying *SMAD4* mutations responsible for Myhre syndrome (MS) [reported by Piccolo et al, (Piccolo et al., 2014)], a condition with large clinical overlap to GPHYSD (Caputo et al., 2012; Le Goff et al., 2012) (Figure 1a and supplementary Figure 1 in Data S1). Immunogold with anti‐LAMP‐1 antibody on fibroblasts from subject 1 showed that the inclusions were decorated by LAMP‐1 signals, thus suggesting that they are enclosed within lysosomes (Figure 1b). Compared to wild type controls, fibroblasts from subject 1 also showed a significantly increased number of LAMP‐1–positive vesicles (Figure 1c) and upregulation of several lysosomal genes (Figure 1d). Incubation of wild type fibroblasts with TGF‐β in the medium resulting in the upregulation of TGF‐β target genes (*COL1A1* and *TIMP1*) showed neither evidence of intracellular storage on EM analysis nor changes in lysosomal gene expression by qPCR (Supplementary Figure 2a‐b in Data S1), thus making unlikely that increased TGF‐β bioavailability is directly involved in the formation of intracellular inclusions.

**Figure 2:**
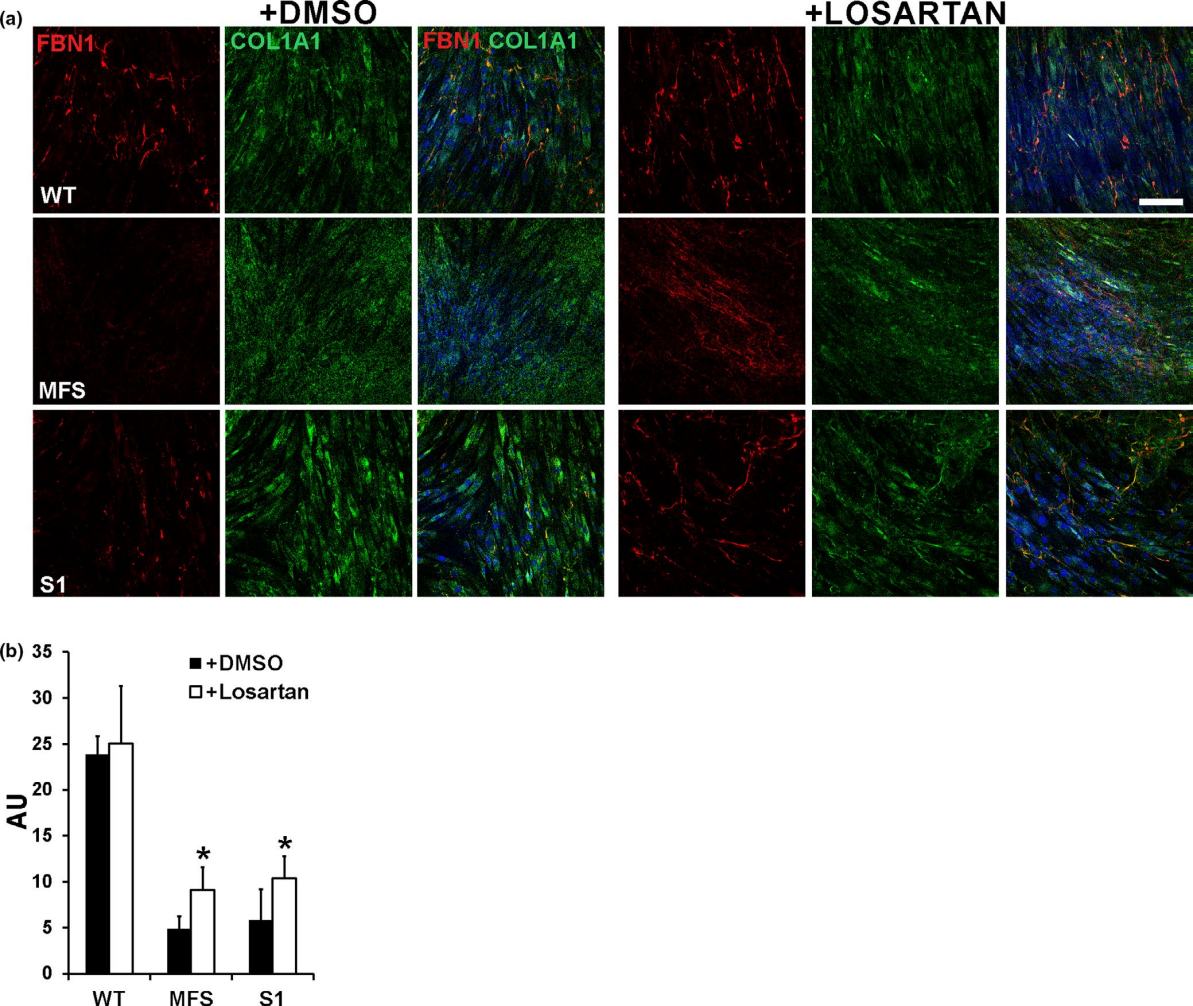
Losartan improves microfibril deposition defect in GPHYSD fibroblasts. Fibroblasts from GPHYSD and MFS patients and from healthy controls (WT) were treated for 14 days with vehicle (DMSO) or 200 μM losartan. (a) Staining for FBN1 *(red)* revealed improved microfibril deposition in losartan‐treated GPHYSD (subject 1, S1) and MFS fibroblasts carrying the p.Asp2104Ter variant in *FBN1*, compared to vehicle‐treated (DMSO) cells. COL1A1 *(green)* staining is shown as ECM deposition control and appears to be not affected by treatment. Nuclei were counterstained with DAPI (blue). Magnification: 20×. Scale bar: 100 μm. (b) Quantification of fluorescence intensity showing significant increase in FBN1 microfibrils deposition after losartan treatment in GPHYSD and MFS fibroblasts. Ratio between FBN1 fluorescence intensity and fluorescence area is expressed as arbitrary units (AU). Averages ± *SEM* are shown; *p < 0.05; *t *test

GPHYSD fibroblasts carrying *FBN1* mutations have reduced and poorly organized ECM microfibrils (Le Goff et al., 2012). In a previous work, we showed that the angiotensin II type 1 receptor inhibitor losartan used to treat aortic aneurysm and prevent dissection in MFS (Brooke et al., 2008), improved microfibril deposition in MS fibroblasts (Piccolo et al., 2014). Therefore, we investigated whether losartan can improve ECM deposition also in GPHYSD carrying *FBN1* mutations. Compared to vehicle‐treated controls, both GPHYSD and MFS fibroblasts incubated with losartan showed improved microfibril deposition as shown by FBN1 staining (Figure 2a,b). Losartan treatment had no effect on lysosomal inclusions evaluated by EM (Supplementary Figure 2c in Data S1).

## 5 DISCUSSION

GPHYSD is a connective tissue disorder of an expanding group of diseases caused by defects of TGF‐β signaling (Doyle, Gerber, & Dietz, 2012). Because intracellular inclusion bodies were previously detected in cells of affected patients, GPHYSD was suspected to be a lysosomal storage disorder (Lipson et al., 1987; Rosser et al., 1995). Here, we confirmed that storage material accumulates within lysosomes in GPHYSD skin fibroblasts and is associated with increased expression of lysosomal genes. Interestingly, we also found that similar inclusions are detected in MS fibroblasts. Nevertheless, as previously shown (Hubmacher, Wang, Mecham, Reinhardt, & Apte, 2015), there is no direct relationship between increased TGF‐β bioavailability and generation of inclusions. Therefore, significance of such inclusions in the pathogenesis of the disease remains unclear.

In the present study, we confirmed that GPHYSD fibroblasts carrying *FBN1* mutations exhibit few and shortened microfibrils that lack a detectable network organization (Le Goff et al., 2011) with a pattern similar to MS fibroblasts (Piccolo et al., 2014). Interestingly, losartan significantly improved microfibril deposition in GPHYSD fibroblasts, like in MFS and MS fibroblasts (Piccolo et al., 2014). Therefore, losartan has potential for treatment of the ECM defect in GPHYSD due to *FBN1* mutations in addition to MS. However, the disease manifestations which can be improved by losartan need to be addressed in preclinical mouse studies and clinical trials. Nevertheless, being an approved and well‐tolerated drug, losartan holds premise for therapy of GPHYSD.

## CONFLICT OF INTEREST

The authors declare no conflict of interest.
