# Supplementary material for: Skin fibroblasts of patients with geleophysic dysplasia due to FBN1 mutations have lysosomal inclusions and losartan improves their microfibril deposition defect
Source: Mol Genet Genomic Med. 2019 Jul 27;7(9):e844. doi: 10.1002/mgg3.844 (PMC6732269; doi:10.1002/mgg3.844)
Supplement: Supplementary file 1 [file MGG3-7-e844-s001.docx]

**Supplementary online material**

*Skin fibroblasts of patients with geleophysic dysplasia due to FBN1 mutations have lysosomal inclusions and losartan improves their microfibril deposition defect.*

Pasquale Piccolo^1,2^, Valeria Sabatino^1^, Pratibha Mithbaokar^1^, Elena Polishchuk^1^, John Hicks^3^, Roman Polishchuk^1^, Carlos A. Bacino^4^, and Nicola Brunetti-Pierri^1,2^

^1^Telethon Institute of Genetics and Medicine, Pozzuoli, Italy; ^2^Department of Translational Medicine, Federico II University of Naples, Naples, Italy; ^3^Department of Pathology, Baylor College of Medicine, Houston, TX, USA; ^4^Department of Molecular and Human Genetics, Baylor College of Medicine, Houston, TX, USA.

**
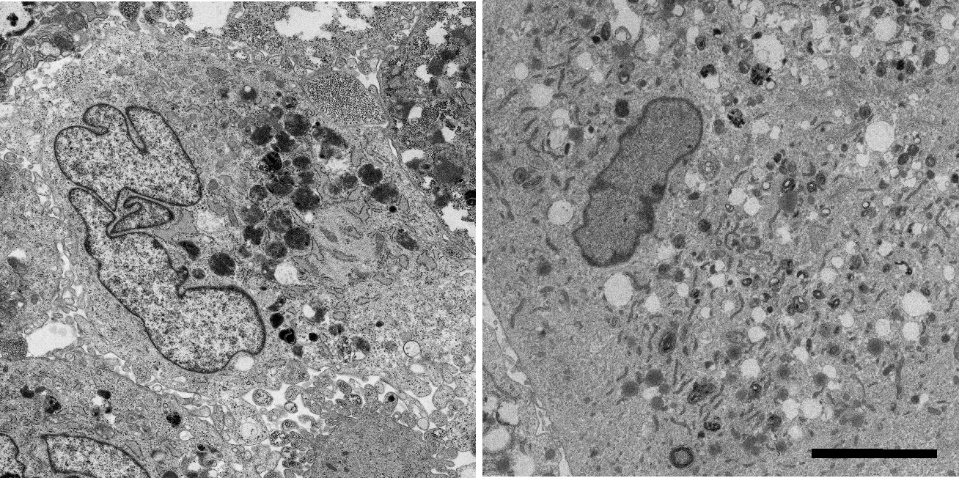
**

**Supplementary Fig. 1. Intracytoplasmic inclusions in Myhre syndrome (MS) fibroblasts.** Electron microscopy analysis on MS fibroblasts showing intracytoplasmic inclusions similar to those observed in GPHYSD. Fibroblasts from subjects 1 and 2 in Piccolo et al, 2014 carrying the p.Ile500Val and p.Arg496Cys *SMAD4* mutation are presented in left and right panel, respectively. Scale bar: 500µm.

**
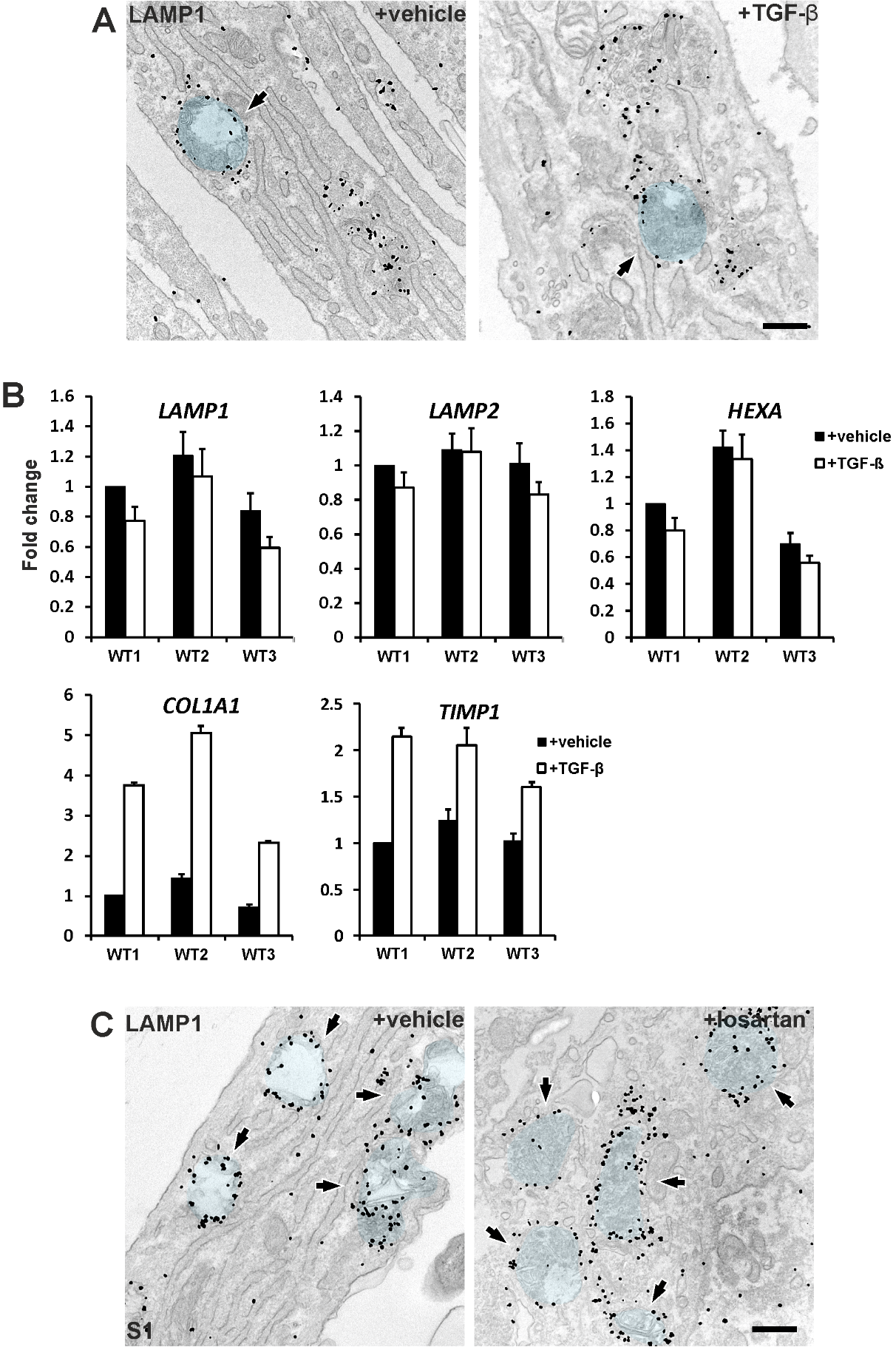
**

**Supplementary Fig. 2. TGF-β is not directly involved in formation of intracellular inclusions. (A)** Representative LAMP-1 immunogold staining of fibroblasts from healthy controls treated for one week with TGF- β (1ng/mL) or vehicle showing no intracellular inclusions. Scale bar: 500nm. **(B)** qPCR in fibroblasts from healthy controls treated for 48 hours with TGF-β (10ng/mL) or vehicle showing no significant changes in lysosomal gene expression (*LAMP1*, *LAMP2*, *HEXA*). Expression of TGF-β transcriptional targets *COL1A1* and *TIMP1* is shown as control of TGF-β activation (t-test: *p*<0.05 +vehicle vs. +TGF-β for *COL1A1* and *TIMP1*). **(C)** LAMP-1 immunogold staining in subject 1 (S1) fibroblasts treated with losartan showing storage within LAMP-1 decorated lysosomal vesicles. Scale bar: 500nm.

**Supplementary Table 1: real time PCR primers**

| **Gene** | **Forward** | **Reverse** |
| --- | --- | --- |
| *COL1A1* | CCCGGGTTTCAGAGACAACTT | TCCACATGCTTTATTCCAGCAATC |
| *CTSB* | AGTGGAGAATGGCACACCCTA | AAGAAGCCATTGTCACCCCA |
| *CTSD* | AACTGCTGGACATCGCTTGCT | CATTCTTCACGTAGGTGCTGGA |
| *CTSF* | ACAGAGGAGGAGTTCCGCACTA | GCTTGCTTCATCTTGTTGCCA |
| *HEXA* | CAACCAACACATTCTTCTCCA | CGCTATCGTGACCTGCTTTT |
| *LAMP1* | ACGTTACAGCGTCCAGCTCAT | TCTTTGGAGCTCGCATTGG |
| *LAMP2* | GCACAGTGAGCACAAATGAGT | CAGTGGTGTGTATGGTGGGT |
| *MCOLN1* | TTGCTCTCTGCCAGCGGTACTA | GCAGTCAGTAACCACCATCGGA |
| *TIMP1* | CTGTTGTTGCTGTGGCTG | CCGTCCACAAGCAATGAG |
| *TPP1* | GATCCCAGCTCTCCTCAATACG | GCCATTTTTGCACCGTGTG |
| *B2M* | GGAGGCTATCCAGCGTACTCC | GGATGGATGAAACCCAGACAC |
| *HPRT1* | TGGCGTCGTGATTAGTGATG | AACACCCTTTCCAAATCCTCAG |
